# Improving Water, Sanitation and Hygiene Practices, and Housing Quality to Prevent Diarrhea among Under-Five Children in Nigeria

**DOI:** 10.3390/tropicalmed3020041

**Published:** 2018-04-12

**Authors:** Sanni Yaya, Alzahra Hudani, Ogochukwu Udenigwe, Vaibhav Shah, Michael Ekholuenetale, Ghose Bishwajit

**Affiliations:** 1 Faculty of Social Sciences, School of International Development and Global Studies, University of Ottawa, Ottawa, ON K1N 6N5, Canada; sanni.yaya@uottawa.ca (S.Y.); ocudenigwe@gmail.com (O.U.); shahvv@icloud.com (V.S.); 2 Population Health, Interdisciplinary School of Health Sciences, University of Ottawa, Ottawa, ON K1N 6N5, Canada; ahuda023@uottawa.ca; 3 Department of Epidemiology and Medical Statistics, Faculty of Public Health, College of Medicine, University of Ibadan, Ibadan 200284, Nigeria; mic42006@gmail.com

**Keywords:** diarrhea, living arrangement, Nigeria, under-five children, water and sanitation

## Abstract

Sub-Saharan Africa as a region accounts for the bulk of the global under-five mortality rate, to which diarrhea is major contributor. Millions of children die from diarrheal diseases each year and those who survive often do so facing suboptimal growth. Preventing the common pathways of transmission for diarrhea-causing pathogens, including improved water, sanitation, and hygiene (WASH) are regarded as the most cost-effective measures for tackling this life-threatening disease. This study aimed to quantitatively assess the quality of living arrangement and access to WASH, and their impact on diarrheal outcomes among under-five children in Nigeria. **Methods**: Data were collected from the 2013 Nigeria Demographic and Health survey (NDHS). Study participants included 28,596 mother-child pairs. Household construction material for wall, floor, and ceiling, access to electricity, and improved water and toilet, were included as the main explanatory variables. Data were analyzed using descriptive and multivariable regression methods. **Results**: The prevalence of diarrhea was 11.3% (95% CI = 10.2–12.6), with the rate being markedly higher in rural (67.3%) as compared to urban areas (32.7%). In the regression analysis, lacking access to improved toilet and water facilities were associated with 14% and 16% higher odds, respectively, of suffering from diarrhea as compared to those who had improved access. **Conclusion**: There is evidence of a weak, but statistically significant, relationship between the quality of living environment, including water and sanitation facilities, and diarrhea among under-five children in Nigeria. The study concludes that investing in living conditions and WASH may have potential benefits for child mortality prevention programs in the country.

## 1. Introduction

Nigeria’s under-five mortality rate is ranked as the eighth highest in the world, and diarrhea is known to be a major contributor to this statistic [1]. Diarrhea is defined as the passage of three of more loose or liquid stools in a 24-h period [2]. The disease is usually a symptom of an intestinal tract infection that is caused by a host of bacterial, viral, and/or parasitic organisms [2]. Diarrhea is considered to be one the leading causes of infant and childhood death in developing countries, and is responsible for 11% of all under-five deaths worldwide [3]. Approximately 19% of total child deaths globally are attributed to diarrheal disease, which affects about 1.87 million children under the age of five [4]. Diarrhea is largely prevalent in developing countries, such as those in sub-Saharan Africa, where there is a lack of safe water, improper means of human fecal waste disposal, intense crowding of rudimentary houses, and poor overall standards of hygiene [5]. As a result of these poor living conditions, young children under the age of five are at a higher risk of being exposed to pathogens that cause diarrhea, which then contributes to a considerable burden of disease within the population. According to the Nigeria Demographic and Health survey (NDHS) (2013), the prevalence of childhood diarrhea in Nigeria lies at about 11%, affecting thousands of children below the age of five [6]. Within the country, diarrhea prevalence is highest in the North Eastern state of Yobe at 35%, and lowest in the Southern states of Bayelsa, Edo, and Ogun, at 2% each [6]. Although there is no indication of the seasonal incidence in the NDHS, prior research illustrates that the incidence of diarrhea among under-five children was the highest during the peak of the dry season (February and April), and the lowest during the rainy season (May–October) [7]. This highly preventable disease causes severe dehydration, which then results in nutritional deficits and/or malnourishment, which can then lead to impaired child growth or death [8]. As a result, diarrhea adversely impacts child growth, fitness, cognition, and performance at school. Additionally, it is estimated that each diarrheal episode that is experienced by a child before his or her second birthday increases the risk of being stunted by five percent [9]. Furthermore, experiences of diarrheal disease in early childhood are associated with long-term adverse cognitive effects and decreased work productivity later in life [10]. Research has shown that poor, unhealthy housing environments increases the risk of infectious diseases, and this is strongly reflected in the nature of the United Nations sustainable development goals.

The United Nations High-Level Political Forum (HLPF) on Sustainable Development has been working with various global stakeholders towards meeting various sustainable development goals each year. In 2017, the HLPF focused on seven goals that would eradicate poverty and promote prosperity in a changing world. One of these seven goals was sustainable development goal (SDG) three, good health and well-being [11]. The SDG progress report (2017) for SDG three reported that a major risk factor for infectious diseases, such as diarrhea and mortality as a result of these diseases is the lack of safe water, sanitation, and hygiene services (WASH) [12]. The lack of safe WASH services disproportionately affects populations living in sub-Saharan Africa and central/southern Asia. In sub-Saharan Africa particularly, the death rates owing to unsafe WASH services, were 46 per 100,000 people [12]. In 2012, approximately 889,000 people died from infectious diseases, such as diarrhea, which was caused largely by fecal contamination in water, as well as inadequate hand-washing practices as a result of non-existent sanitation facilities [12]. Furthermore, the report indicates a considerably higher mortality rate for children under the age of 5 in sub-Saharan Africa at 84 deaths per 1000 live births in comparison to global statistics, which reports 43 deaths per 1000 live births. The HLPF agenda for 2018 is intended to focus on ‘transformation towards sustainable and resilient societies’ [11]. SDG Six, access to safe water and sanitation, is one of the six goals that was included in the 2018 HLPF agenda, and is directly related to what is needed in Nigeria to reduce under-five mortality that is associated with diarrheal disease.

Unfortunately, cases of diarrhea are still widely prevalent in Nigeria, despite various government-led interventions that have been implemented to tackle the issue [13]. Diarrheal diseases are largely preventable, and more action at various levels of government and civil society organizations within Nigeria are necessary to reduce the associated under-five mortality rate. The most common cause of childhood diarrhea in Nigeria is due to the contamination of food and water by germs or infectious organisms that proliferate in poor living conditions [2]. In fact, the lack of safe water, basic sanitation, and hygiene account for about 88% of the disease burden due to diarrhea [14]. A large proportion of literature has focused on curative strategies to tackle this infectious disease among sick children, such as appropriate community home management and the use of oral rehydration solutions to rehydrate them [8,15]. However, more preventative solutions are needed to reduce the associated morbidity and mortality rates. This study examines the association between household characteristics, such as toilet facilities, water quality, access to electricity, and the existence and quality of walls, roofs, and flooring in Nigerian dwellings, and diarrhea amongst various under-five children in Nigeria. NDHS 2013 data have been thoroughly analyzed using descriptive and multivariate regression methods.

## 2. Methods

### 2.1. Data Source

Data for this study are derived from the Nigerian Demographic and Health Survey (2013). These surveys are delivered by the National Population Commission, which receives financial and technical assistance from the Inner City Fund (ICF) International that is provisioned through the USAID-funded MEASURE DHS program. National DHS surveys consist of data on a wide range of public health topics including anthropometric, demographic, socioeconomic, family planning, and domestic violence issues, to name a few. The NDHS (2013) provides up-to-date information on the socio-demographic characteristics of the respondents between the ages of 15–49 from randomly selected households and children under-five who reside in non-institutional settings. For sampling, a three-staged stratified cluster design was employed, which was based on a list of enumeration areas (EAs) from the 2006 Population Census of the Federal Republic of Nigeria. EAs are systematically selected units from the localities, which constitute the local government areas (LGAs). LGAs are subdivisions of each of the 36 administrative states (including the Federal Capital Territory, called Abuja) and are classified under six developmental zones in the country. EAs were used to form the survey clusters, called primary sampling units. Furthermore, fieldwork was conducted from 15 February 2013, to the end of May (with the exception of the two teams in Kano and Lagos, who completed fieldwork in June). A more detailed version of the survey was published elsewhere [6].

### 2.2. Study Variables

The outcome variable was the prevalence of diarrhea during the two weeks prior to household survey administration. Mothers were asked whether or not their child suffered from diarrhea during the past two weeks, and had the options of answering either as ‘Yes’ or ‘No’.

The main independent variables comprised living arrangement characteristics that were proxied by the following indicators: access to electricity, improved sanitation, improved water, children’s excreta disposal facilities, and construction material for wall, floor, and ceiling. For construction materials, the indicators were categorized as concrete (cement, bricks, stone with lime, ceramic tiles) or not concrete (made up of earth, sand, mud, cardboard, wood plank, bamboo). Guidelines from the Joint Monitoring Program developed by the WHO and UNICEF, were used to classify the type of water and the sanitation facilities as either improved or unimproved (Table 1) [16].

To adjust the analysis for potential confounders, the following variables were included, based on their theoretical relevance to the outcome variable.

Age of child: 0–12/13–24/25–36/37–48/49–59; Sex: Female/Male; Stunted: No/Yes; Residency type: Urban/Rural; Religion: Christianity/Islam/Other; Mother’s education: No education/Primary/Secondary/Higher; Has insurance: No/Yes; Fathers education: No education/Primary/Secondary/Higher; No. of children in HH: 1–2/3–5/>5; Wealth index: Poorest/Poorer/Middle/Richer/Richest.

### 2.3. Data Analysis

Before the dataset was analyzed, it was checked for missing values and outliers. To adjust for the cluster sampling techniques of the surveys, a complex survey module was used for all of the analyses by accounting for primary sampling units, sample strata, and sample weight. Following that, descriptive analyses were carried out to calculate the prevalence rates of diarrhea. Chi-square tests were performed to examine the bivariate association between diarrhea and the explanatory variables. Variables that were found to be significant at alpha 5% were entered into the regression analysis [17]. Binary logistic regression analyses were carried out to calculate the odds ratios of the association between diarrhea and various living arrangement characteristics, while adjusting for socio-demographic variables. The level of significance was set at alpha 5% for the regression models. All of the analyses were performed using SPSS version 24 for Windows.

### 2.4. Ethical Consideration

Before participating in the interview, all of the participants provided informed consent to the surveyors. DHS surveys are also approved by ICF international as well as an institutional review board (IRB) in the host country to ensure that the protocols are in compliance with the U.S. Department of Health and Human Services regulations for the protection of human subjects.

## 3. Results

### 3.1. Socio-Demographic Findings

In total, 28,596 mothers between the ages of 15–49 years participated in the survey. Basic socio-demographic characteristics of the participants are summarized in Table 2. The reported prevalence of diarrhea among under-five children was 11.3% within the two weeks prior to data collection. Cases of under-five diarrhea were significantly higher in rural areas of residence in comparison to urban areas of residence, with 67.3% of cases being situated in urban regions and 32.7% in rural ones. Additionally, parental levels of education and their position within the wealth index also played a significant role in whether or not their under-five children acquired diarrhea. Results also indicated that the higher the education level of both mother and father, and the wealthier the family, the less likely the child or children from that household would be to acquire diarrhea. Therefore, children with both an uneducated mother and father are at a significantly higher risk of diarrhea (55% and 48.5%, respectively, *p* < 0.0001) in comparison to those who have primary, secondary or higher education, and those who are poorest are also at highest risk. Furthermore, the study found that stunted children were at higher risk of acquiring diarrhea in comparison to non-stunted children (64.7% vs. 35.3%, *p* < 0.0001). Of all the socio-demographic factors that were investigated, the sex of the child was the only variable not associated with under-five diarrheal outcome.

Statistically significant associations were observed between diarrhea and physical household environments, such as access to electricity, improved water and toilet facility, children’s excreta disposal facilities, as well as having concrete walls, flooring, and roofing. Results show that children living in households with no electricity (57.7% vs. 42.3%, *p* < 0.0001), unimproved water quality (51.1% vs. 48.9%, *p* < 0.0001) or toilet facilities (51.7% vs. 48.3%, *p* = 0.033), are more likely to suffer from diarrhea. Furthermore, having concrete walls, floor, and roof also influenced diarrheal outcomes for under-five children that are living in these households.

### 3.2. Multivariate Regression Analysis

Results of the multivariate regression analysis are summarized in Table 3. In the first step of the multivariate regression analysis model (Model 1), diarrhea was regressed only against the household living arrangement factors, which include toilet and water facilities; and, building material for household walls, roof, and floor. Partially adjusting for these characteristics revealed a significant predictability of electricity; toilet and water facilities; and, roof material. After adjusting for individual level factors (i.e., demographic factors, such as age, sex, and stunting) in Model 2, the predictability of water facility remained significant; however, that of toilet facility was no longer significant. Model 3 adjusted for religion and type of residence, and it had no visible change in the predictability of water and roof quality. In the fully adjusted model (Model 4), all of the variables appeared to be significant predictors of diarrhea in under-five children, except for the children’s excreta disposal facilities, and the building material of wall. Households with no access to electricity had 27% higher odds [OR = 1.269; 95% CI = 1.053–1.529] of reporting diarrhoea than those who had access to electricity. Not having access to improved toilet and water facilities were associated with 14% [OR = 1.14, 95% CI = 1.040–1.250] and 16% [OR = 1.160, 95% CI = 1.064–1.265] higher odds of diarrhea, respectively, as compared to those who had improved access to these facilities. Children in households without concrete roofing and walls also had 16% [OR = 1.164, 95% CI = 1.046–1.294] and 14% [OR = 1.140, 95% CI = 1.040–1.250] higher odds of diarrhea, respectively, when compared to those that were concrete.

## 4. Discussion

Identifying and implementing effective and sustainable solutions to prevent diarrhea, which is a life-threatening infectious disease, is crucial to achieve SDG three and to improve child survival by the year 2030. This study aims to investigate the association between family household environments including access to safe and adequate WASH, and diarrheal outcomes in under-five children in Nigeria, based on the NDHS 2013. The sample description indicates that a majority of households in Nigeria live in rural environments, are not educated, and do not have insurance [6]. Young children among these families are at a higher risk of morbidity and mortality from infectious diseases, such as diarrhea. It is not surprising to see evidence from several other Nigerian studies reflecting the deep influence of various intersecting social determinants of health, such as level of education, socioeconomic status, and physical environment playing a significant role in the adverse outcomes that are experienced by vulnerable populations, such as under-five children [12,18,19].

The NDHS (2013) comprises a large and representative sample size of 28,596 study participants from Nigeria. Additionally, the survey non-response rate was less than 10%, which is favorably low [20]. However, the study does have some drawbacks. First, at the time of data collection, mothers were asked whether their child or children had diarrhea at anytime during the two weeks preceding the survey. The validity of this indicator is affected by the mother’s perception of what diarrhea is, as well as her capacity to recall the event. As well, the mothers’ ability to recall the event does not provide any information about the duration or severity of the child or children’s diarrheal episodes. Additionally, the prevalence of diarrhea does vary seasonally, and therefore the results of the NDHS findings may be impacted by this factor [6]. Furthermore, more information is needed on the household characteristics that are described in Table 2, as the quality of toilet and water facilities (i.e., improved or unimproved) can vary by level. This information is necessary to inform relevant program and policy development that is aiming to reduce under-five morbidity and mortality as a result of diarrhea.

Based on the analysis of the results, there is evidence of a weak, but statistically significant relationship between poor living environments and diarrhea among under-five children. Prior literature identifies poor water quality and toilet facilities as critical risk factors predisposing under-five children to diarrhea. The NDHS (2013) shows a marked difference between stool disposals according to the various background characteristics of the sample. The national survey also indicates that the safe and proper disposal of children’s feces is highly important in preventing the spread of disease, as direct contact with human feces can cause diarrhea and/or other related infectious diseases. Therefore, it is crucial that toilet facilities are improved, especially in the rural regions within Nigeria, to prevent unnecessary contact with children’s feces during use of, or disposal into, a toilet or latrine [6]. Furthermore, there is more evidence from Nigerian studies that shows many avenues through which water quality deteriorates at the household level. Some of these avenues include poor, unhygienic water handling and poor storage of drinking water [13,21]. Additionally, a study by Oloruntoba and colleagues (2014), found that six specific WASH related risk factors predisposed under-five children to a higher incidence of diarrhea, which included: poor drinking water-handling; lack of hand-washing with soap after defecating and before food preparation [21]; clogged drainage around or in close proximity to the place of residence; breeding places for flies or other insects near the house; and, total overall hygiene practices. It is important to note that all of these factors can amplify without sufficient housing environments and access to adequate WASH facilities in the home environment. Furthermore, a recent study (2017) emphasized the need to strengthen policies related to WASH practices such as unsanitary disposal of child feces, which can contribute to the spread of diarrhea [22].

A report by the World Bank (2010), investigating WASH and children’s health based on evidence from 172 DHS surveys from 70 countries, including those in sub-Saharan Africa over the last 25 years demonstrates that child morbidity and mortality from diarrhea is substantially lower for those that have access to more ‘advanced’ WASH technologies, such as flushed toilets with piped connections in comparison to ‘basic’ WASH technologies, such as latrines or a lack of access to any WASH technology [23]. Study findings show that access to water and sanitation technologies reduces the odds of suffering from under-five child diarrhea by 7.3% and 12.9%, respectively [23]. Similar reductions are also found in the associated under-five mortality risk, which therefore indicates the existence of the Mills-Reinke Multiplier [23]. Additionally, fixed estimates from this review indicate that children in households with access to a flush toilet show 17% lower odds for diarrhea than those using open defecation. This World Bank report, in addition to three reviewed meta-studies, found that improved sanitation has a somewhat higher positive effect on diarrhea than water infrastructure [24,25,26]. Therefore, there is strong evidence that more resources need to be invested in improving sanitation from no access or basic sanitation technologies to advanced sanitation technologies in Nigeria.

As far as we are aware, this is the first study to report on WASH practices and household conditions and its association with diarrhea among under-five children in Nigeria. Although the data were cross-sectional, they were large enough to help make meaningful conclusions, as the samples were collected nationwide. Several important indicators including construction material and access to improved water and sanitation facilities were used to assess the quality of household environments. One of the main limitations to this study includes the self-reported nature of the variables that incurs the risks of recall and reporting biases, and the lack of information on medications, and the presence of other diseases that could have influenced the strength of the association to certain degrees. Furthermore, the survey was cross-sectional, and hence no causal relationship can be established from the findings. The outcomes of the study need to be further assessed by longitudinal studies, with the inclusion of a wider range of enteric diseases.

Improving WASH in community housing may potentially be the most cost-effective preventative solution in decreasing the burden of disease that is associated with diarrhea and under-five mortality in the country. In Nigeria, there is generally a low level of knowledge about appropriate WASH practices, as well as inappropriate practices performed among caregivers on the different aspects of home management of childhood diarrhea [8,15]. However, if the exposure to diarrhea-causing pathogens is significantly reduced through adequate WASH in the household environment, then there will be a lessened need to turn to home-based treatment and management. As a result, it is recommended that public and private organizations work collaboratively to develop a comprehensive strategy that addresses the inadequate WASH practices among families. They should also work to improve and advance household infrastructure, such as water quality and toilet facilities, which influence these WASH practices in Nigerian households. As well, there is a need for more financial, material, and human resources to be invested in educating families through community-based programming, and supporting them in making the necessary changes, both physically in their houses and behaviorally with regards to relevant WASH practices. Implementing these strategies may improve overall household environments, and may thereby prevent the associated under-five childhood morbidity and mortality that is caused by diarrhea.

## 5. Conclusions

Based on the analysis of the NDHS, this study concludes that substandard living conditions can contribute to the increasing burden of diarrhea among under-five children in Nigeria. Statistically significant and positive associations were observed between diarrhea in the two weeks prior to survey data collection, with having no access to electricity, improved water and sanitation, and concrete structure of households. In addition to household factors, important socio-demographic and regional disparities were found in the prevalence of diarrhea. Based on these findings, it is recommended that health policy decision-makers pay special attention to investing in the living conditions and WASH, especially among the disadvantaged communities in Nigeria, with an emphasis on those that are residing in the rural North. This, in the long run, might contribute to reduced child morbidity and mortality from diarrhea in the country.

## Figures and Tables

**Table 1:** Joint Monitoring Program classification of improved sanitation and water supply [16].

	Unimproved	Improved
Sanitation	Unimproved sanitation facilities do not ensure hygienic separation of human excreta from human contact. Unimproved facilities include pit latrines without a slab or platform, hanging latrines and bucket latrines.	Improved sanitation facilities ensure hygienic separation of human excreta from human contact. They comprise of the following facilities: Flush/pour flush to: piped sewer system, septic tank, pit latrine; ventilated improved pit (VIP) latrine, pit latrine with slab, composting toilet.
Water	Unimproved drinking-water sources include: Unprotected dug well, unprotected spring, cart with small tank/drum, surface water (river, dam, lake, pond, stream, canal, irrigation channels), and bottled water.	Improved drinking-water sources include: Public taps or standpipes, tube wells or boreholes, protected dug wells, protected springs or rainwater collection.Piped water on premises: Piped household water connection located inside the user’s dwelling, plot or yard.

**Table 2:** Socio-demographic profile of the study participants (NDHS, 2013).

	N = 28,596	%	OR	95% CI Lower	95% CI Upper	*p*-Value
**Age of child**						
0–12	6235	21.8	22.4	20.8	24.0	<0.001
13–24	5834	20.4	31.1	29.1	33.1	
25–36	5384	18.8	20.2	18.6	22.0	
37–48	5669	19.8	14.3	12.8	15.8	
49–59	5474	19.1	12.1	10.9	13.3	
**Sex**						0.471
Female	14,209	49.7	49.9	49.2	50.6	
Male	14,387	50.3	50.1	49.4	50.8	
**Stunted**						<0.0001
No	9294	32.5	35.3	33.1	37.4	
Yes	19,302	67.5	64.7	61.9	67.5	
**Residency type**						<0.0001
Urban	9685	33.9	32.7	29.4	36.3	
Rural	18,911	66.1	67.3	63.7	70.6	
**Religion**						<0.0001
Christian	11,697	40.9	28.5	25.4	31.7	
Islam	16,467	57.6	69.7	66.4	72.9	
Other	432	1.5	1.8	1.2	2.6	
**Mother’s education**						<0.0001
No education	13,105	45.8	55.0	51.7	58.2	
Primary	5836	20.4	18.3	16.4	20.3	
Secondary	7818	27.3	23.5	21.1	26.2	
Higher	1837	6.4	3.2	2.5	4.1	
**Has insurance**						0.031
No	27,971	97.8	98.2	97.5	98.6	
Yes	625	1.8	1.8	1.9	2.2	
**Father’s education**						<0.0001
No education	11,191	39.1	48.5	44.9	52.0	
Primary	5408	18.9	19.0	17.1	21.0	
Secondary	8345	29.2	25.4	23.0	27.9	
Higher	3652	12.8	7.2	5.9	8.8	
**Number of children in household**						<0.0001
1–2	18,977	66.4	64.6	62.2	66.9	
3–5	9117	31.9	33.4	31.3	35.7	
>5	502	1.8	2.0	1.4	2.8	
**Wealth index**						<0.0001
Poorest	6241	21.8	27.7	24.4	31.3	
Poorer	6499	22.7	25.8	23.4	28.3	
Middle	5770	20.2	18.8	16.5	21.2	
Richer	5394	18.9	15.4	13.2	17.9	
Richest	4692	16.4	12.3	10.4	14.6	
**Household has electricity**						<0.0001
No	14,804	51.8	57.7	53.6	61.7	
Yes	13,792	48.2	42.3	38.3	46.4	
**Water quality**						<0.0001
Unimproved	15,346	53.7	51.1	47.0	55.2	
Improved	13,250	46.3	48.9	44.8	53.0	
**Toilet facility**						0.033
Unimproved	14,596	51.0	51.7	48.1	55.3	
Improved	14,000	49.0	48.3	44.7	51.9	
**Children’s excreta disposal facility**						<0.0001
Unimproved	11,095	38.8	39.3	37.4	41.2	
Improved	17,501	61.2	60.7	58.8	62.6	
**Concrete wall**						<0.0001
No	5505	19.3	16.7	14.1	19.7	
Yes	23,091	80.7	83.3	80.3	85.9	
**Concrete floor**						<0.0001
No	16,089	56.3	47.4	43.9	50.9	
Yes	12,507	43.7	52.6	49.1	56.1	
**Concrete roof**						0.004
No	1066	3.7	3.2	2.4	4.3	
Yes	27,530	96.3	96.8	95.7	97.6	

N.B. *p*-values are from chi-square tests.

**Table 3:** Association between household characteristics and diarrhea among under-five children in Nigeria [6].

	Model 1			Model 2			Model 3			Model 4		
	OR	95% CI Upper	95% CI Lower	OR	95% CI Upper	95% CI Lower	OR	95% CI Upper	95% CI Lower	OR	95% CI Upper	95% CI Lower
Electricity (Yes)												
No	**1.463**	1.358	1.577	**1.468**	1.362	1.582	**1.278**	1.184	1.380	**1.269**	1.053	1.529
Toilet (Improved)												
Unimproved	**1.155**	1.065	1.253	1.079	0.989	1.178	1.095	1.002	1.196	**1.140**	1.040	1.250
Children’s excreta disposal facility (Improved)												
Unimproved	**1.149**	0.968	1.363	1.102	0.904	1.342	1.042	0.748	1.452	**1.071**	0.758	1.511
Water (Improved)												
Unimproved	**1.131**	1.046	1.223	**1.180**	1.084	1.284	**1.174**	1.079	1.278	**1.160**	1.064	1.265
Wall (concrete)												
No	1.053	0.949	1.167	**1.138**	1.013	1.278	1.123	0.999	1.263	1.081	0.959	1.218
Roof (concrete)												
No	**1.377**	1.265	1.498	**1.264**	1.155	1.383	**1.274**	1.162	1.397	**1.164**	1.046	1.294
Floor (concrete)												
No	1.076	0.862	1.341	0.969	0.734	1.278	**1.095**	1.002	1.196	**1.140**	1.040	1.250

N.B. Reference categories are shown in parenthesis. Model information: Model 1: Electricity, Toilet, children’s excreta disposal facilities, water, Wall, floor and roof material; Model 2: Model 1, Age, Sex, Stunting; Model 3: Model 2, residency, religion; Model 4: Mothers education, insurance, father’s education: CI = Confidence Interval. OR = Odds ratio. ORs in bold are significant at *p* < 0.05.
